# Sex in aging matters: exercise and chronic stress differentially impact females and males across the lifespan

**DOI:** 10.3389/fnagi.2024.1508801

**Published:** 2025-01-15

**Authors:** D. Gregory Sullens, Kayla Gilley, Luke E. Moraglia, Sarah Dison, Jessica T. Hoffman, Madison B. Wiffler, Robert C. Barnes, Annie T. Ginty, Melanie J. Sekeres

**Affiliations:** ^1^Department of Psychology and Neuroscience, Baylor University, Waco, TX, United States; ^2^Department of Biology and Chemistry, Liberty University, Lynchburg, VA, United States; ^3^Department of Psychology, The University of Texas at Dallas, Richardson, TX, United States; ^4^Department of Biology, Baylor University, Waco, TX, United States; ^5^Department of Neurobiology, University of Utah, Salt Lake City, UT, United States; ^6^Department of Pharmacology and Neuroscience, Texas Tech University Health Sciences Center, Lubbock, TX, United States; ^7^School of Psychology, University of Ottawa, Ottawa, ON, Canada

**Keywords:** aging, sex differences, running, stress, memory, anxiety-like behavior, hyperactivity

## Abstract

Assessing sex as a biological variable is critical to determining the influence of environmental and lifestyle risks and protective factors mediating behavior and neuroplasticity across the lifespan. We investigated sex differences in affective behavior, memory, and hippocampal neurogenesis following short- or long-term exposure to exercise or chronic mild stress in young and aged mice. Male and female mice were assigned control, running, or chronic stress rearing conditions for 1 month (young) or for 15 months (aged), then underwent a behavioral test battery to assess activity, affective behavior, and memory. Stress exposure into late-adulthood increased hyperactivity in both sexes, and enhanced anxiety-like and depressive-like behavior in aged female, but not male, mice. One month of stress or running had no differential effects on behavior in young males and females. Running increased survival of BrdU-labelled hippocampal cells in both young and aged mice, and enhanced spatial memory in aged mice. These findings highlight the importance of considering sex when determining how aging is differently impacted by modifiable lifestyle factors across the lifespan.

## 1 Introduction

Attention to the need to consider biological sex differences across the lifespan has increased in recent years, with the majority of neurobiological research involving models of the brain and aging being conducted using only males (Beery and Zucker, 2011; Klein et al., 2015; Beltz et al., 2020; Rechlin et al., 2022). Differences in affective and cognitive processing between biological sex is well documented, with males generally performing better at spatial-related cognitive processing, and females having a higher prevalence of stress, anxiety and fear-related affective disorders (Bauer, 2023; Yagi and Galea, 2019). At the neural level, molecular mechanisms within the central amygdala and hippocampus have been shown to differentially regulate fear memory consolidation and extinction in young male and female mice (Florido et al., 2024; Matsuda et al., 2015; Velasco et al., 2019). Morphologically, sex differences within the hippocampus have been observed in young rodents and regulated by estrous cycling, including gross hippocampal volume (Qiu et al., 2013), dendritic spine density (Woolley et al., 1990), and neural cell proliferation (Rummel et al., 2010). Confounding factors and variability associated with active estrous cycling in females is often given as the rationale for using exclusively male rodents in studies of cognition and physiology (Becegato and Silva, 2024; Kaluve et al., 2022), limiting and biasing the pool of studies of brain and behavior across the lifespan. A recent meta-analysis of studies assessing preclinical behavioral models of anxiety-like behavior found that just 21% of studies included both sexes and considered biological sex in their analyses, and that 61% of published studies used exclusively male subjects (Kaluve et al., 2022). While a growing number of studies are considering how environmental factors such as stress exposure during early-life and critical developmental periods differentially impact males and females (Bale and Epperson, 2015; Guadagno et al., 2018; Hodes and Epperson, 2019; LoPilato et al., 2020), direct investigations of sex differences in behavior and brain plasticity in response to modifiable environmental and lifestyle risk factors and protective factors active into late-adulthood are scarce (Bangasser and Cuarenta, 2021).

Adequately assessing differences in cognitive and affective behavior along with brain structure and function across the lifespan in humans exposed to different environmental factors remains a considerable challenge. Many studies tend to be correlational, rely on retrospective self-report, are limited to measures of observable forms of brain activity with limited spatial and temporal resolution, or are based on post-mortem examination of brain pathology (Grady, 2012; Palliyaguru et al., 2021). While these approaches provide valuable insight, they are limited in identifying underlying changes at the cellular level (Fischer, 2016). Rodent models of aging can overcome this limitation and provide critical information about differences in neuronal plasticity in the young and aged brain, while maintaining control over exposure to external environmental factors. However, due to cost-prohibitive limitations on aging research using animal models, few studies to date have directly investigated sex differences related to the aging brain and behavior (Palliyaguru et al., 2021; Özalay et al., 2024).

Chronic stress has been associated with detrimental effects to both physical and psychological health (McEwen and Gianaros, 2010; O’Connor et al., 2021; Sapolsky, 1996). These effects include memory impairment (Kim and Diamond, 2002; Luine et al., 2017) and increased levels of anxiety and depression (McEwen, 2004). Further, it has been shown that behavioral stress is associated with increased inflammatory glial responses (Frank et al., 2007; Bollinger et al., 2017), pro-inflammatory cytokine expression (Mograbi et al., 2020), depleted brain neurotrophic growth factors (Duman and Monteggia, 2006; Murakami et al., 2005; Notaras and van den Buuse, 2020), stunted prefrontal cortex dendritic growth (Bloss et al., 2010), and reduced hippocampal volume and neurogenesis (Duman, 2004; Jung et al., 2020; Schoenfeld et al., 2017). Many of the adverse effects of stress exposure compound with age and may contribute to an acceleration of brain aging (Bishop et al., 2010; Wolf et al., 2024; Yegorov et al., 2020).

Certain modifiable lifestyle and environmental factors may be protective against age-related brain and behavioral changes. Brief periods of exposure to voluntary aerobic exercise and environmental enrichment contribute to beneficial effects on neuronal development and cognition, including enhanced hippocampal-dependent memory and hippocampal neurogenesis (Epp et al., 2021; Kempermann et al., 1997, 2002; Scholz et al., 2015; van Praag et al., 1999a, 1999b, 2005), increases in endothelial growth factors (Cao et al., 2004; Fabel et al., 2003; Morland et al., 2017) and brain-derived neurotrophic factor (BDNF), particularly in young adults (Adlard et al., 2005; Cotman et al., 2007; Huang et al., 2014; Wrann et al., 2013; Maass et al., 2016). There is strong evidence that long-term engagement in voluntary aerobic exercise such as running is neuroprotective, and protective against cognitive decline with aging in humans (Chan et al., 2005; Duzel et al., 2016; Tyndall et al., 2018) and rodents (Alvarez-López et al., 2013; Diederich et al., 2017; Robison et al., 2018; Connolly et al., 2022). However, the degree to which running confers protection against cognitive impairment and promotes brain plasticity may differ in males and females (Triviño-Paredes et al., 2016; Barha et al., 2017a, 2017b; Barha and Liu-Ambrose, 2018; Loprinzi and Frith, 2018). One study exposing mice to daily voluntary running throughout young and middle adulthood did include both male and female subjects, but analyses considering biological sex were not performed (Robison et al., 2018). A systematic review of sex differences in aerobic exercise effects on brain and cognition using aged rodent models noted that most studies conducted to date used only a single sex, the majority of which were male, limiting any conclusions that can be directly drawn related to the effectiveness of exercise exposure in aged males and females (Barha et al., 2017b).

In male mice, 16 months of continuous exposure to voluntary wheel running enhanced synaptic plasticity-related gene expression in the hippocampus and improved spatial memory relative to sedentary sex-matched controls, suggesting a cumulative benefit of aerobic exercise throughout the lifespan in aged males (Stranahan et al., 2010). Aged male mice exposed to 2 months of late-life voluntary running exhibited enhanced spatial memory performance relative to socially isolated aged mice, although neither of these late-life environmental factors (running or isolation) significantly altered gene expression of microglial and astrocytic genes (i.e., Iba1, GFAP) or anti-inflammatory cytokines in the hippocampus (Ederer et al., 2022), suggesting that relatively brief environmental changes in late-life may not robustly alter brain physiology in males, despite being sufficient to impact spatial memory and behavior. Findings on the effects of environmental enrichment including voluntary running on brain and cognition in aged females are also mixed. Late-life exposure to 6 months of voluntary running decreased anxiety-like behavior in the open field, enhanced spatial memory, and increased rates of late-adult hippocampal neurogenesis and BDNF levels in aged female mice, suggesting that periods of prolonged exercise in late-adulthood are beneficial to affect, cognition, and brain plasticity in females (Marlatt et al., 2012). However, female mice continuously exposed to enrichment conditions, including access to voluntary wheel running for 11 months, showed enhancements in memory performance, despite no enhancements in neurogenesis (Hüttenrauch et al., 2016). The apparent inconsistency in brain and behavior between sexes in response to brief or prolonged enrichment and exercise into late-adulthood is striking, yet the methodological differences used across studies make it difficult to directly compare their findings.

Exposure to chronic mild stress has differential effects on males and females (Bremner and Narayan, 1998; Lupien et al., 2009; McLaughlin et al., 2009), although the majority of animal model investigations considering the role of sex in reactivity to chronic stress involve juvenile and young adult animals with stressor periods typically lasting only 1–8 weeks (Franceschelli et al., 2014). In response to 21 days of chronic restraint stress, young adult female rats showed decreased dendritic branching complexity and length within hippocampal CA3 subfield, and increased corticosterone relative to males experiencing the same restraint stress (Galea et al., 1997), suggesting that the female brain is particularly sensitive to the effects of chronic stress exposure in young adulthood. At the behavioral level, young adult female rats have shown greater resilience to chronic restraint stressors than young adult male rats, which show impairments in spatial and non-spatial memory (Bowman et al., 2003, 2009). The same sex distinction was not observed in aged rats exposed to 3 weeks of restraint stress in late-adulthood, with comparable memory performance in both males and females on the spatial recognition test that was unimpaired by stress (Bowman et al., 2006). Potential sex differences following chronic stress exposure throughout the entire adult lifespan are still unknown. These findings highlight the importance of identifying how environmental factors may differentially impact males and female behavior and neural physiology across the lifespan and between biological sexes.

As there are no studies contrasting behavior and brain plasticity in male and female mice with identical environmental rearing conditions into late-adulthood, it is not yet known the degree to which life-long chronic stress and exercise differentially impact cognition, affect, and brain plasticity between biological sexes. A central goal of this study is identifying how modifiable environmental factors (running, stress) may differentially influence affective and cognitive behavior and brain plasticity in males and females across the lifespan. Given the co-morbidity seen with late-life inactivity, depression, and dementia (Alexopoulos, 2005; Cunningham et al., 2020; Strawbridge et al., 2002), understanding the impact of environmental risk factors is critical to developing interventions aimed at protecting against age-related cognitive decline, anxiety and depression, neurodegeneration and other pathologies that may differentially affect males and females. Here, we investigated sex differences on affective behavior, memory, and hippocampal cell division related to the effects of long-term exposure to chronic mild stress or voluntary exercise in young and aged male and female mice.

## 2 Methods

### 2.1 Subjects

**Figure 1:**
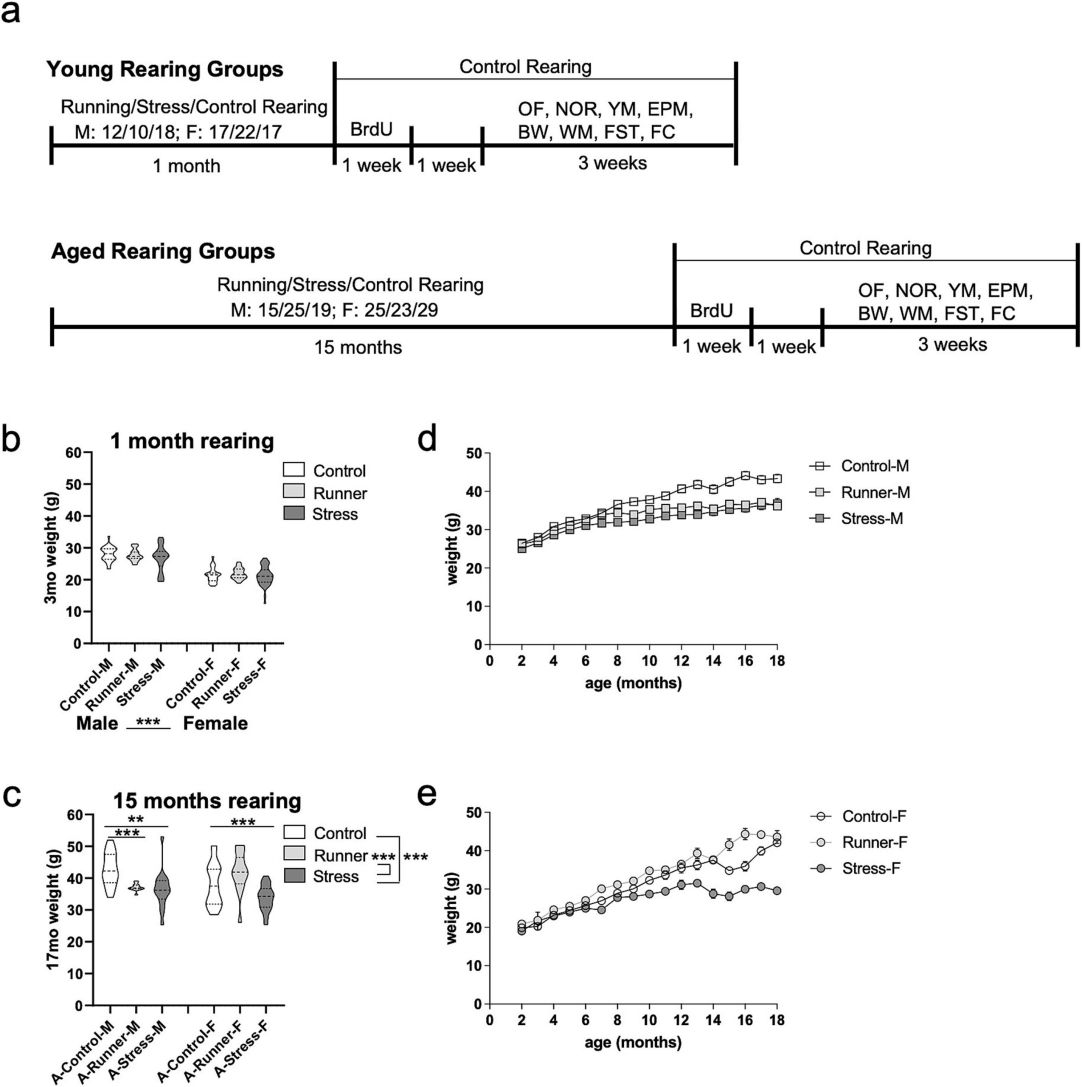
Experimental timeline and weight record. **(A)** Schematic of the study timeline. Male and female mice were assigned to either a running, stress, or control rearing condition for 1 month (top, young) or 15 months (bottom, aged). Following the assigned rearing period, all mice were placed under the control rearing condition and given BrdU treatment (2 injections per day for 7 days) followed by a 7 day rest period, and 3 weeks of post-rearing behavioral testing including: open field (OF; 1 trial), novel object recognition task (NOR; sample trial, 24-h delay test trial; 2 days), Y-maze (YM; sample trial, 2 min delay test trial; 1 day), elevated plus maze (EPM; 1 trial; 1 day), beam walk (BW; 3 trials, 1 day), spatial water maze training (WM; 5 trials/day; 5 days), forced swim task (FST; 1 trial; 1 day), and fear conditioning (FC; conditioning trial, 24-h delay context test trial and tone test trial; 2 days). Mouse weights (grams, g) at after 1 month **(B)** and 15 months **(C)** of rearing. Weight records throughout the rearing and testing period for aged male **(D)** and female **(E)** mice. Error bars represent the standard error of the mean (SEM). ^**^p ≤ 0.01 and ^***^p ≤ 0.001.

Female and male F1 hybrid C57BL/6J (Jackson Labs) × 129S6/SvEvTac (Taconic) were bred in the Baylor University rodent vivarium. At 8 weeks of age, mice were randomly assigned to one of three rearing conditions: control rearing (control), running rearing (runner), or chronic mild stress rearing (stress). Mice were further subdivided into two aging conditions (young, aged). Mice assigned to the young condition were reared for 1 month and mice assigned to the aged condition were reared for 15 months, under their assigned rearing conditions (*n* = 232, group *n*’s = 10–29; see Supplementary Table S1 for groups). Following rearing of 1 or 15 months, all mice were placed under control rearing conditions for the remainder of the study in order not to impact behavioral testing. Mice received 5-bromo-2′-deoxyuridine (BrdU) injections to label proliferating cells. Two weeks post-injections, mice underwent a battery of post-rearing behavioral testing for 3 weeks. Mice were weighed each month throughout the rearing period and checked daily for visual signs of distress (piloerection, hunched posture, lethargy). Any mouse exhibiting signs of distress, poor body condition, or lost >20% body weight during the rearing period were removed from the study (15 mice lost to attrition). See Figure 1A for a schematic of the study design and timeline. All procedures were approved by Baylor University’s Institutional Care and Use Committee and conducted in accordance with the Guide for the Care and Use of Laboratory Animals from the National Institutes of Health.

### 2.2 Rearing conditions

#### 2.2.1 Control rearing (control)

Mice were group-housed with sex-matched conspecifics (3–5 per cage) in standard polysulfone cages (19.9 cm × 37.9 cm × 13.0 cm; Tecniplast, USA) with corncob bedding and cotton batting nesting materials, using ventilated racks. Mice had *ad libitum* access to food and water and were maintained at an ambient temperature of 22 ± 2C°, on a standard 12 h light–dark cycle (lights on 600 h–1,800 h), with behavioral testing conducted during the light phase of the cycle.

#### 2.2.2 Running rearing (runner)

Mice were reared under the control housing conditions, with unlimited access to a metered low-profile running wheel (Fast Trac wheel, 15.24 cm diameter, Bio Serv) in the home cage. Digitally recorded data (distance travelled, time of activity) was sent to the wireless running wheel USB interface hub (Med Associates).

#### 2.2.3 Chronic mild stress rearing (stress)

Mice were group-housed in standard polysulfone cages with corncob bedding. Mice had *ad libitum* access to food and water throughout the study, except during limited restriction periods described below. No enrichment stimuli were placed in the home cage. All mice in the stress group were exposed to a series of chronic mild stress manipulations throughout the rearing period. Mice were exposed to 5 stressors per week of rearing. Mice received a single stress manipulation (food or water restriction, light cycle manipulation, restraint, social isolation, confined social approach, or wet cage) on a given day (see Supplementary Table S2 for sample schedule of stressors for 1 month). The number of weekly stressors was selected based on other chronic mild stress paradigms, which typically consist of exposing mice to 2–7 unpredictable social and non-social stressors per week over the course of multiple weeks (Willner, 2017; Burstein and Doron, 2018). These manipulations have all been previously used as stress models in rodents (Duman and Monteggia, 2006).

##### 2.2.3.1 Food or water restriction

For food or water restriction, access was removed for 23 h, after which *ad libitum* access resumed. Food or water restriction sessions never exceeded a combined total of 3 non-consecutive restriction sessions per week. Food and water restriction are considered ecologically valid conditions for inducing mild chronic stress (Willner, 1997), as it mimics the stressors and pressures of the animal’s natural environment. Weekly weight records were kept for all mice in the stress condition to ensure their weight did not drop below 80% of their free-feeding weight.

##### 2.2.3.2 Light cycle manipulation

Mice were relocated to a separate colony room and exposed to 36 h of light or 36 h of darkness, after which they were transferred back to resume rearing under the normal 12 h light–dark cycle.

##### 2.2.3.3 Restraint

Mice were individually restrained in a clear Plexiglas, flat-bottom restrainer with ventilation holes (3.81 cm width × 7.62 cm length × 2.54 cm height, Plas-Labs) for 1 h.

##### 2.2.3.4 Social isolation

Mice were single-housed in a standard polysulfone cage with corncob bedding for 8 h, with *ad libitum* access to food and water.

##### 2.2.3.5 Confined social approach

The mouse was confined to a cylinder cage (7.62 cm diameter, 17.78 cm height, metal bars spaced 0.64 cm apart). The cage was placed within a rectangular compartment (43.18 cm width × 20.32 cm length × 22.86 cm height). An unfamiliar conspecific mouse was placed in the chamber with the cage and allowed to explore the cage containing the confined mouse for 10 min (Toth and Neumann, 2013).

##### 2.2.3.6 Wet cage

Two hundred and fifty milliliters of water was poured onto the bedding in the home cage, and left for 8 h (Li et al., 2007). Mice were then returned to a clean home cage with fresh bedding.

### 2.3 Behavioral testing

Behavioral testing began 2 weeks following the end of the experimental rearing period for both young and aged groups in order to accommodate BrdU injections and recovery prior to initiating behavioral testing. Mice were handled individually by an experimenter for 5 min/day for 5 days prior to beginning behavioral testing. A post-rearing behavioral test battery assessed affective behavior (anxiety-like and depressive-like behavior) and memory (see Figure 1A for test battery details and test order). The behavioral test battery was conducted over the course of 3 weeks, with only a single behavioral task conducted each day. See Figure 1A for behavioral testing timeline. Behavioral tasks were conducted in a least-to-most aversive order, and each test apparatus was cleaned with 70% ethanol between trials. Mice were returned to their home cage following completion of each behavioral task. For all behavioral tasks, activity was recorded by a digital camera and activity levels were analyzed using the SMART video-tracking system (Panlab, RRID:SCR_002852) software, unless otherwise stated.

#### 2.3.1 Tasks to assess affective behavior

##### 2.3.1.1 Open field

The open field task was used to assess exploratory and anxiety-like behavior (Prut and Belzung, 2003). The open field arena (45 cm × 45 cm × 40 cm) consisted of four white, solid acrylic walls. Mice were placed individually in the center of the arena and allowed to freely explore for 15 min. The number of entries and percentage of time spent in the different zones of the open field (periphery, middle, and center), and the distance and speed travelled were analyzed. The periphery zone is defined as the area within 10 cm from the edge of the wall, and the center zone is defined as the inner-most 7.5 × 7.5 cm area. Preference for the periphery zone is indicative of a thigmotaxic, anxiety-like phenotype (Seibenhener and Wooten, 2015).

##### 2.3.1.2 Elevated plus maze

The elevated plus maze (EPM) task was also used as a measure of anxiety-like behavior, with less time spent in the open arms of the maze indicating an anxiety-like phenotype (Hogg, 1996). The white Plexiglas apparatus consisted of 2 open arms (30 cm long × 5 cm wide × 0 cm high) and 2 closed arms (30 cm long × 5 cm wide × 15 cm high) extended from a central platform (5 cm × 5 cm) elevated 50 cm above the floor. Mice were individually placed on the central platform facing an open arm and allowed to freely explore the maze for 5 min. The latency (seconds) to enter a closed arm of the maze, the total time spent in open and closed arms of the maze, and the distance and speed travelled were measured.

##### 2.3.1.3 Beam walk

A beam (2 cm wide, 30 cm long) was elevated 50 cm above the ground, with a 10 × 10 × 10 cm dark escape chamber attached to the end of the beam. A bright light was placed at the start end of the beam to encourage the mouse to move towards the escape chamber at the opposite end. The mouse was placed at the start end of the beam and the latency to enter the escape chamber was recorded by a blinded experimenter using a stopwatch. If a mouse failed to enter the escape chamber, it was assigned the maximal 300 s score. Mean escape latency scores across 3 trials were calculated for each mouse.

##### 2.3.1.4 Forced swim task

A clear Plexiglas cylinder (10 cm diameter, 25 cm high, Panlab) was filled halfway with room temperature water. The mouse was placed in the chamber for a single 6 min test and immobility behavior during the task was recorded. Immobility was defined as a lack of movement other than the minor movement required to maintain the head above water (Castagné et al., 2011). After completion of the trial, mice were dried with a towel and placed back in the home cage, which was partially located on heating pad. Lower periods of activity are indicative of a learned helplessness phenotype or a stress-coping strategy (Porsolt et al., 1977; Cryan et al., 2002; Commons et al., 2017).

#### 2.3.2 Tasks to assess cognition

##### 2.3.2.1 Y-maze

The Y-maze task is a measure of spatial recognition memory, with preference for the novel arm of the apparatus being indicative of memory performance (Conrad et al., 1996; Kraeuter et al., 2019). The grey Plexiglas Y-maze apparatus consisted of three arms (30 cm long × 9 cm wide × 15 cm high) extended from a common central platform (8 cm × 8 cm × 15 cm). For the sample trial, one arm of the Y-maze was blocked by a removable door. The mouse was placed on the central platform facing an open arm and allowed to explore the 2 accessible arms of the maze for 5 min. The mouse was returned to a holding cage for a 2 min inter-trial interval. The door blocking access to the third arm of the maze was then removed, and the mouse was again placed on the central platform facing an open arm and allowed to freely explore for an additional 3 min for recognition the test trial. The latency to enter the novel arms of the maze, the number of entries into each arm, and the speed and distance travelled were measured.

##### 2.3.2.2 Novel object recognition

The novel object recognition (NOR) task is a well-established measure recognition memory based on a rodent’s preference for novelty (Eichenbaum et al., 2007). An open field arena, as described previously, was used to conduct both the acquisition and the test phase of the NOR task. During the NOR acquisition phase, an object (e.g., LEGO blocks, toy car) was placed on one side of the arena. Mice were individually placed in the center of the arena and allowed to explore the object for 5 min before being returned to their home cage. Twenty-four hours later, mice were returned to the chamber, and both the familiar object used during acquisition and a similar sized novel object were presented in different locations of the chamber. Novel and familiar objects were counterbalanced across mice. Exploratory behavior of each object during the 5 min test was measured. Exploration was defined as the mouse’s time spent in proximity to the object (≤1 cm), with its head facing the object (Knapman et al., 2010).

##### 2.3.2.3 Spatial water maze

Spatial memory was assessed using the Morris water maze (Morris et al., 1982). A circular plastic pool (127 cm diameter) was filled to a depth of 40 cm with water (~26°C) made opaque by the addition of nontoxic paint. A circular escape platform (10 cm diameter) was submerged 0.5 cm below the surface of the water in the center of one of the pool quadrants. The pool was surrounded by distinct geometric cues located on the walls of the room. During training, mice were given 5 non-consecutive trials per day for 5 days, with a 30 min inter-trial interval. Each trial started with the mouse being released into the pool from one of four possible points, facing the wall. The order of release points varied pseudo-randomly across days. The trial ended when the mouse mounted the platform with all four paws, or after 60 s elapsed. If a mouse failed to find the platform, it was guided by the experimenter. At the end of each trial, the mouse was allowed to rest atop the platform for 15 s. After completion of the trial, mice were dried with a towel and placed back in the home cage partially located on heating pad. For each trial, the latency to mount the platform was recorded by a blinded experimenter using a stopwatch. Mean escape latency scores across the 5 trials per day were calculated for each mouse.

##### 2.3.2.4 Tone and context fear conditioning

Fear conditioning was conducted in a chamber (19 cm × 20 cm × 128 cm) with a shock grid floor (bars 3.2 mm in diameter spaced 7.9 mm apart), clear acrylic front and back walls, and aluminum sidewalls and roof (Coulborn Instruments). The mouse was allowed 2 min to explore the chamber, then received 3 foot shocks (0.5 mA, 2 s duration, 1 min apart) paired with a tone (2,800 Hz, 85 dB, 30 s). The mouse was removed from the chamber 1 min after the last shock (Sekeres et al., 2012). The chamber was cleaned with an ethanol solution between trials. Context test: twenty-four hours later, the mouse was replaced in the conditioning chamber and freezing behavior was recorded for 3 min. Tone test: the tone was then sounded for an additional 3 min, and freezing levels were measured. Freezing is a species-specific defense reaction that is typically used as a measure of fear in rodents (Kim and Fanselow, 1992). Behavior in the chamber was recorded by an overhead camera, and activity levels were analyzed using Freezeframe software (Actimetrix, RRID:SCR_014429).

### 2.4 BrdU treatment

Following the end of the rearing period (1 month rearing for young; 15 months rearing for aged), mice received 2 daily i.p. injections (12 h apart) of BrdU (Sigma, 19–160) for 7 consecutive days (100 mg/kg, BrdU dissolved in sterile phosphate buffered saline). Following BrdU injections, mice were given 1 week rest prior to beginning behavioral testing. BrdU is a thymidine analog that is incorporated into newly synthesized DNA, used to measure post-natal cell division in the rodent hippocampus (Wojtowicz and Kee, 2006). Mice were sacrificed 1 month following the final BrdU injection.

### 2.5 Sacrifice and BrdU immunohistochemistry

Eight mice per group were randomly selected for immunohistochemical analysis of BrdU expression in the dentate gyrus. Following behavioral testing, mice were anesthetized using an isoflurane and oxygen mixture and intracardially perfused with PBS and 4% paraformaldehyde (PFA). Brains were removed from the skull, and post-fixed in PFA for 24 h at 4°C then transferred to a 0.02% sodium azide PBS solution until sectioning. Brains were sectioned coronally using a vibratome (Leica, VT1000 S) at 30um. Serial sections were stored 5 sections per well in 0.02% sodium azide-PBS solution. Using a systematic random sampling method, one section per well was sampled between −1.58 to −2.92 A/P from bregma, for a total of 8 sections per brain. Sections were washed in 4 × 10 min PBS, incubated in HCl at 45°C for 45 min for denaturing, washed in 3 × 2 min PBS, incubated with blocking solution at RT for 30 min (1X PBS, 3% Triton X, and 2% normal goat serum), and then incubated with rat-anti-BrdU primary antibody (1:200, PBS and 0.3% Triton X-100, Abcam, ab6326) at 4°C for 48 h. Sections were then washed 4 × 10 min in PBS and incubated with goat-anti-rat Alexa 488 secondary antibody (1:200, Abcam, ab150157) for 2 h at room temperature. Sections were then washed with 3 × 10 min PBS, then mounted with PermaFluor mounting medium on glass slides, and cover slipped.

#### 2.5.1 BrdU quantification

Stained sections were imaged and analyzed using a Nikon Eclipse-NI-E fluorescent microscope. The dentate gyrus upper and lower blades were counted live on the microscope by two investigators blind to experimental conditions. The investigators randomly chose a subset of mice that they both counted to assess inter-rater reliability, with a ~5% margin of error accepted. Data are presented as the number of BrdU-positive cells per section. Cell count criteria were restricted to spherical GFP^+^ cells located within the upper and lower blades of the granule cell layer. Only sections with intact upper and lower blade staining were included in the analyses, and sections corresponding the Paxinos and Franklin (2019) were matched between conditions to ensure equivalent sampling between brains. For each counted section, data are presented for the entire dentate gyrus, although a similar pattern of main effects were observed when assessing the upper and lower blades separately (data not shown, but available upon request). Up to 2 brains per group were excluded from analyses due to poor tissue integrity following immunostaining.

### 2.6 Statistical analyses

Univariate ANOVAs were conducted for behavioral measures and BrdU analyses. Post-hoc *t*-tests of Tukey’s HSD tests were used to assess significant main effects and interactions. All statistical analyses were conducted using SPSS 26 (RRID:SCR_002865) by an experimenter blind to experimental condition. Effects were considered significant at *p* < 0.05. Where appropriate, Bonferroni corrections for multiple comparisons were applied. Data are represented in the figures using truncated violin plots, with the dark dotted line indicating the group median and the fine dotted lines indicating the upper and lower quartiles for each condition (GraphPad 12, Prism; RRID:SCR_002798). Due to technical issues related to video and software malfunction, and logistical issues related to scheduling during the pandemic, data from certain mice were unavailable for all behavioral tasks.

## 3 Results

### 3.1 Long-term chronic stress reduces weight in aged mice

One month of running or chronic mild stress exposure was not sufficient to alter the overall body weight of young mice. Long-term running and exposure to chronic mild stress was associated with low body weight of aged mice. A 3 (rearing condition: control, runner, stress) × 2 (sex: male, female) ANOVA was conducted using both the young and aged mice after 1 month of rearing (3 months of age for all mice). A main effect emerged for sex [*F*_(1,195)_ = 242.247, *p* < 0.001, *η*^2^*_p_* = 0.554], with female mice weighing less than males. One month of rearing was not sufficient to differentially affect weight, with no main effect of rearing [*F*_(2,195)_ = 2.506, *p* = 0.084, *η*^2^*_p_* = 0.025], nor a sex × rearing interaction [*F*_(2,195)_ = 0.661, *p* = 0.518, *η*^2^*_p_* = 0.007] (Figure 1B). After 15 months of continuous rearing under the assigned rearing condition in the aged group, ANOVA identified a main effect of rearing condition [*F*_(2,78)_ = 15.893, *p* < 0.001, *η*^2^*_p_* = 0.290] with aged stressed mice weighing less than control (*p* < 0.001) and runners (*p* = 0.001) (Figure 1C). There was no main effect of sex [*F*_(1,78)_ = 0.007, *p* = 0.933, *η*^2^*_p_* = 0.000], but a significant rearing × sex interaction [*F*_(2,78)_ = 5.002, *p* = 0.009, *η*^2^*_p_* = 0.114] in aged mice. Post-hoc analyses using age and sex-matched control-reared mice found that both aged running-reared male [*t*_(23)_ = 3.841, *p* = 0.001, *d* = 1.569] and stress-reared male mice [*t*_(28)_ = 3.223, *p* = 0.003, *d* = 1.987] weighed significantly less than controls (Figure 1D). Similarly, aged stress-reared females [*t*_(30)_ = 3.792, *p* = 0.001, *d* = 1.318] weighed significantly less than controls, while aged running-reared females exhibited comparable weights relative to controls [*t*_(22)_ = −0.705, *p* = 0.488, *d* = 0.303] (Figure 1E). These results suggest that long-term (15 months), but not short term (1 month) exposure to chronic stress results in low weight gain in both sexes.

### 3.2 Assessments of locomotor activity and affect

#### 3.2.1 Open field

**Figure 2:**
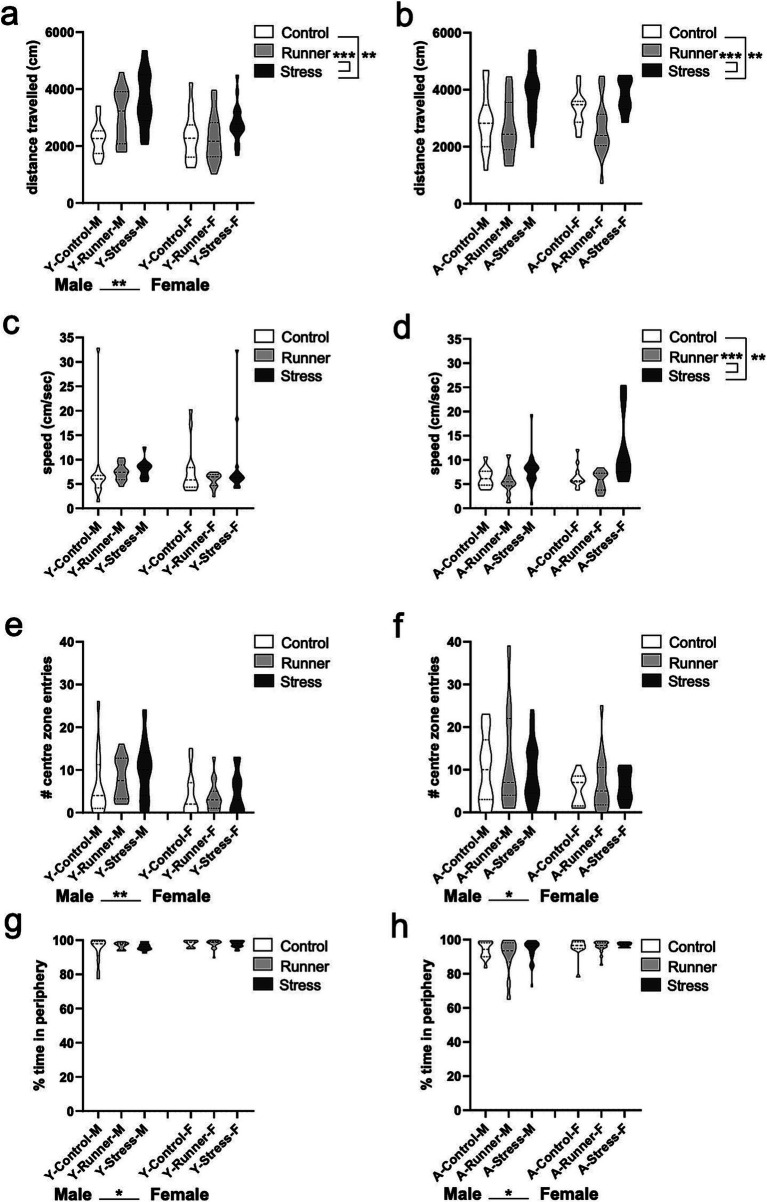
Open field activity. Stressed mice exhibit hyperactive behavior in the open field (OF) arena. Distance travelled (cm) (**A**, young; **B**, aged) and speed (cm/s) (**C**, young; **D**, aged) during the OF task. Young and aged female mice exhibit more anxiety-like behavior than aged-matched males. The number of entries into the center zone of the open field (**E**, young; **F**, aged) and the percentage of time in the periphery of the OF arena (**G**, young; **H**, aged). ^*^p < 0.05, ^**^p ≤ 0.01, and *p* < 0.001.

When examining the total locomotor activity within the open field arena, a main effect of rearing condition emerges for young [*F*_(2,79)_ = 9.036, *p* < 0.001, *η*^2^*_p_* = 0.186] (Figure 2A) and aged mice [*F*_(2,77)_ = 10.640, *p* < 0.001, *η*^2^*_p_* = 0.217], and a main effect of sex for young mice [*F*_(1,79)_ = 8.415, *p* = 0.005, *η*^2^*_p_* = 0.096] (Figure 2B). Tukey’s post-hoc test confirms that both young and aged mice reared under stress conditions travel a greater distance than control-reared mice (young, *p* = 0.002; aged, *p* = 0.005) and running-reared mice (young, *p* < 0.001; aged, *p* < 0.001). A similar main effect of rearing condition was observed for travelling speed in aged mice [*F*_(2,77)_ = 13.179, *p* < 0.001, *η*^2^*_p_* = 0.255] (Figure 2D), but not in young mice [*F*_(2,79)_ = 0.781, *p* = 0.461, *η*^2^*_p_* = 0.019] (Figure 2C), with aged stressed mice moving faster than either control (*p* = 0.003) or runner mice (*p* < 0.001). These findings indicate a hyperactive phenotype in aged stressed mice. When assessing their exploratory behavior, main effects of sex were seen for both young [*F*_(1,79)_ = 10.569, *p* = 0.002, *η*^2^*_p_* = 0.118] (Figure 2E) and aged mice [*F*_(1,77)_ = 6.229, *p* = 0.015, *η*^2^*_p_* = 0.075] (Figure 2F), with males entering the center zone of the arena more frequently than females. Assessment of the total percentage of time spent in the periphery of the open field arena also revealed main effects of sex for young mice [*F*_(1,79)_ = 6.660 *p* = 0.012, *η*^2^*_p_* = 0.078] (Figure 2G) and aged mice [*F*_(1,77)_ = 6.120, *p* = 0.016, *η*^2^*_p_* = 0.074] (Figure 2H), with female mice spending more time in the periphery, indicative of an anxiety-like phenotype, regardless of rearing condition. No further main effects of rearing condition, or rearing × sex interactions were observed (all *p*’s >0.05).

#### 3.2.2 Elevated plus maze

**Figure 3:**
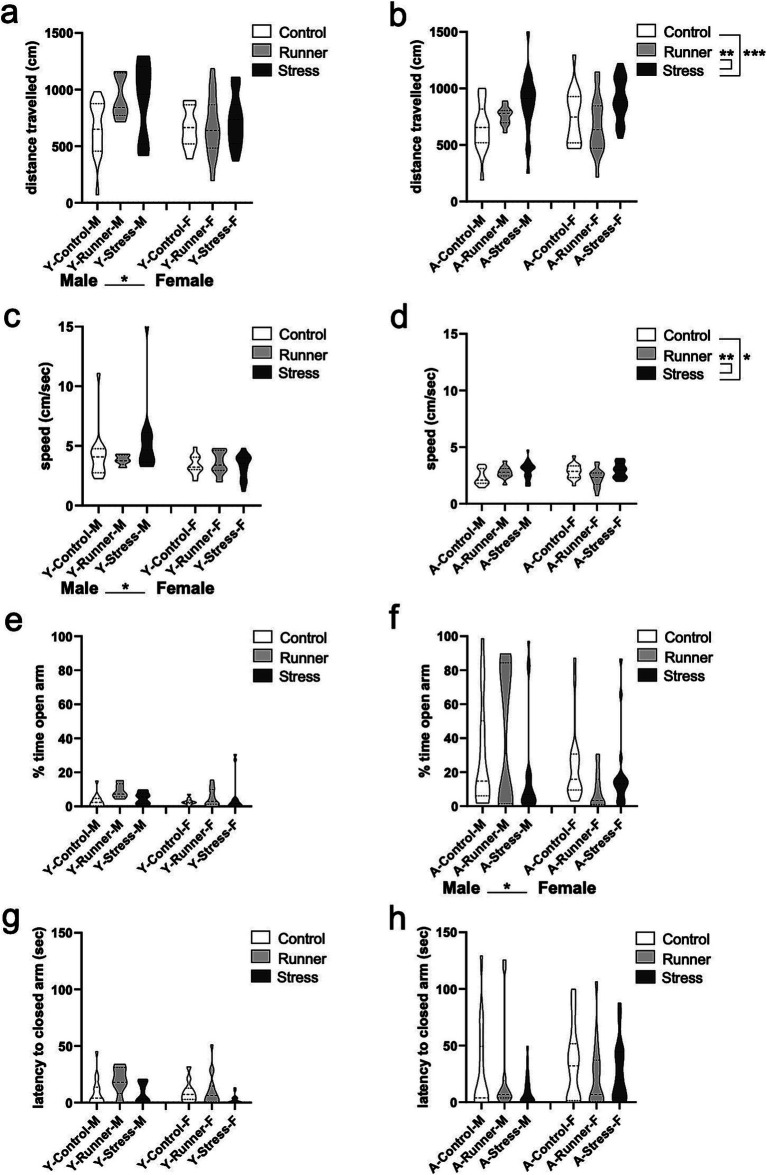
Elevated plus maze activity. Aged stressed mice exhibit hyperactive behavior in the elevated plus maze (EPM). Distance travelled (cm) (**A**, young; **B**, aged) and speed (cm/s) (**C**, young; **D**, aged) during the EPM task. Aged female mice exhibit more anxiety-like behavior than aged-matched males. The percentage of time in the open arms (**E**, young; **F**, aged) and latency (sec) to enter a closed arm of the EPM (**G**, young; **H**, aged). ^*^p < 0.05, ^**^p ≤ 0.01, and ^***^p < 0.001.

A trending main effect of rearing was observed in the distance travelled in young mice [*F*_(2,66)_ = 2.944, *p* = 0.060, *η*^2^*_p_* = 0.082], and a significant main effect of sex [*F*_(1,66)_ = 5.252, *p* = 0.025, *η*^2^*_p_* = 0.074], with young males travelling farther than females (Figure 3A). Consistent with what was observed in the open field, main effects of rearing were found for the total distance travelled in aged mice [*F*_(2,96)_ = 7.636, *p* = 0.001, *η*^2^*_p_* = 0.137], with stressed mice exploring significantly further distances than controls (*p* = 0.001) and runners (*p* = 0.003) (Figure 3B). A main effect of sex on speed was also confirmed in young mice, with males travelling faster than females [*F*_(1,66)_ = 6.869, *p* = 0.011, *η*^2^*_p_* = 0.094] (Figure 3C). A main effect of rearing on speed was observed in aged mice [*F*_(2,96)_ = 8.58, *p* = 0.024, *η*^2^*_p_* = 0.074], with stressed mice moving significantly faster than controls (*p* = 0.038) and runners (*p* = 0.007) (Figure 3D), confirming the hyperactive behavior in aged stressed mice.

No group differences were observed for the percent of time spent in the open arms of the EPM for young mice (all *p*’s >0.05) (Figure 3E), and only a main effect of sex was observed for aged mice [*F*_(1,96)_ = 5.518, *p* = 0.021, *η*^2^*_p_* = 0.054], with females spending less time than males in the open arms of the maze (Figure 3F). No further main effects, or rearing × sex interactions were observed (all *p*’s >0.05). No group differences were observed for the latency to enter the closed arms of the maze for either young (Figure 3G) or aged mice (Figure 3H) (all *p*’s >0.05).

#### 3.2.3 Beam walk

**Figure 4:**
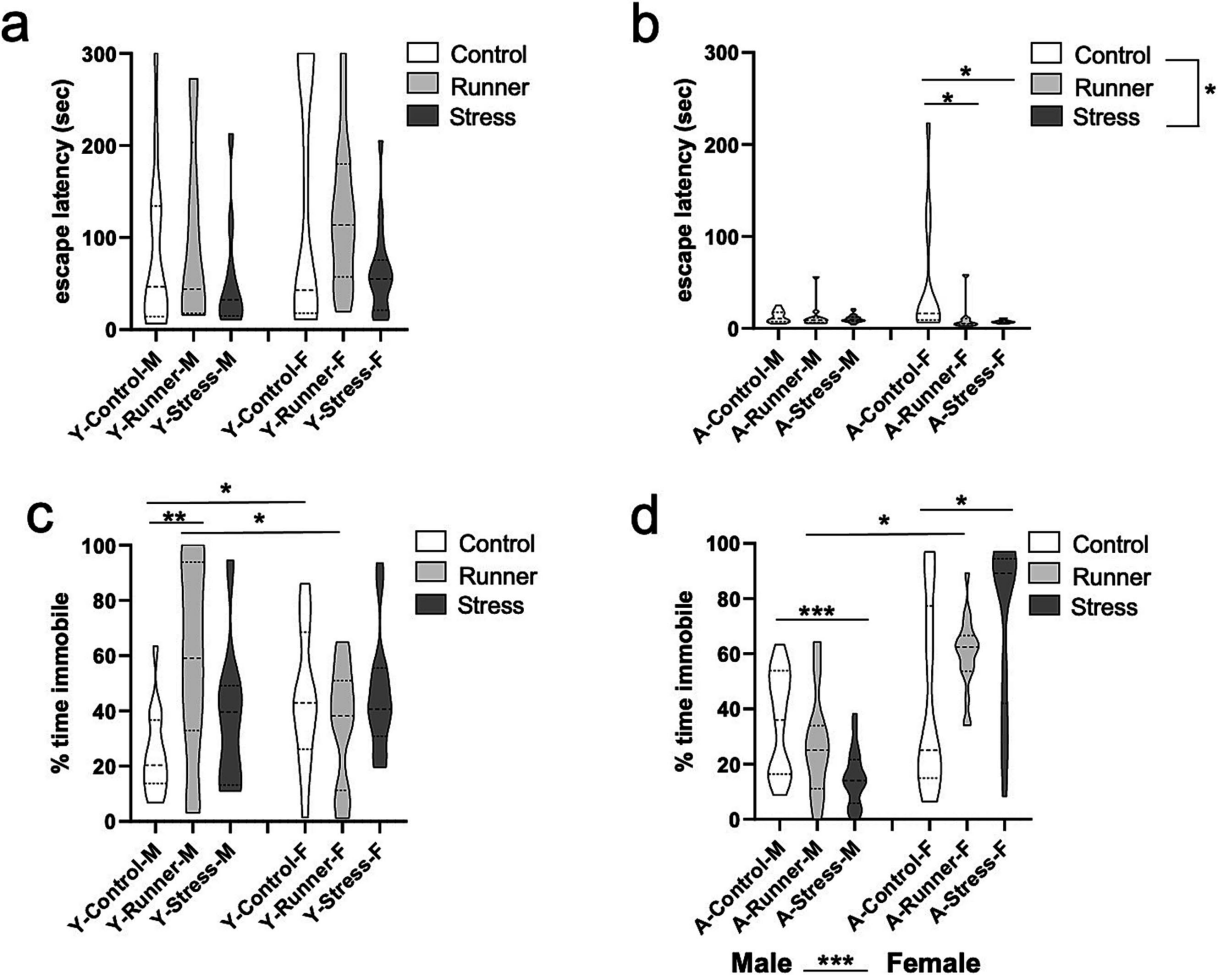
Beam walk and forced swim task activity. Escape latencies (sec) in the beam walk task (**A**, young; **B**, aged). Percent of time spent immobile in the forced swim task (FST) (**C**, young; **D**, aged). ^*^p < 0.05, ^**^p ≤ 0.01, and ^***^p < 0.001.

We assessed escape behavior and motor activity in the beam walk task (Brooks and Dunnett, 2009), with shorter escape latencies into the dark escape chamber being indicative of increased anxiety-like behavior. Aged mice demonstrated a main effect of rearing condition on escape latency [*F*_(2,71)_ = 3.955, *p* = 0.024, *η*^2^*_p_* = 0.100], with stressed mice exhibiting significantly faster escape times than control (*p* = 0.019; Figure 4B). A significant rearing × sex interaction emerged for aged mice [*F*_(2,71)_ = 3.499, *p* = 0.036, *η*^2^*_p_* = 0.090], driven by shorter escape latencies in runner [*t*_(18)_ = 2.398, *p* = 0.032, *d* = 1.025] and stressed females [*t*_(17)_ = 2.751, *p* = 0.019, *d* = 1.156] when compared with sex-matched controls. Young mice did not show these same main effects or interactions (all *p*’s >0.05) (Figure 4A).

#### 3.2.4 Forced swim task

In assessing learned helplessness in the forced swim task (FST), we found that young mice in all rearing conditions spent a comparable percentage of time immobile [*F*_(2,74)_ = 1.910, *p* = 0.115, *η*^2^*_p_* = 0.049, Figure 4C]. No main effect of sex was observed [*F*_(1,74)_ = 0.119, *p* = 0.731, *η*^2^*_p_* = 0.002], but a rearing × sex interaction emerged [*F*_(2,74)_ = 5.890, *p* = 0.004, *η*^2^*_p_* = 0.137, Figure 4C], with young male runners spending more time immobile than young female runners [*t*_(24)_ = 2.087, *p* = 0.048, *d* = 0.806]. The inverse was observed for young female control mice, with female mice spending more time immobile relative to male controls [*t*_(28)_ = −2.707, *p* = 0.011, *d* = 0.989]. Male runners also spent more time immobile relative to male control mice [*t*_(25)_ = −3.449, *p* = 0.002, *d* = 1.281], whereas young female runners spent less time immobile relative to female controls [*t*_(27)_ = 1.068, *p* = 0.295, *d* = 0.372]. These findings suggest that running may reduce learned helplessness in young females, but not young males. Aged mice show a main effect of sex [*F*_(1,95)_ = 49.606, *p* < 0.001, *η*^2^*_p_* = 0.343] in overall immobility behavior, with females displaying more immobility relative to males (Figure 4D). As in young mice, there was no main effect of rearing [*F*_(2,95)_ = 0.388, *p* = 0.608, *η*^2^*_p_* = 0.008], but a rearing × sex interaction was observed [*F*_(2,95)_ = 10.439, *p* < 0.001, *η*^2^*_p_* = 0.180]. Interestingly, prolonged running led to reduced immobility behavior in aged male mice when contrasted with aged-matched female mice [*t*_(23)_ = −2.214, *p* = 0.037, *d* = 0.833]. In aged males, prolonged stress was associated with less immobility behavior relative to control mice [*t*_(35)_ = 4.232, *p* < 0.001, *d* = 1.346]. The opposite pattern emerged in aged female mice, with stress-reared females displaying significantly more immobility behavior than age-matched control females [*t*_(33)_ = −2.610, *p* = 0.014, *d* = 0.884]. This indicates a robust sex difference in the impact of prolonged chronic stress on learned helplessness in aged mice, with stress exacerbating learned helplessness in females, but increasing struggling behavior in males during the inescapable swim task.

### 3.3 Long-term stress or exercise generally does not interfere with memory performance in aged mice

#### 3.3.1 Y-maze

**Figure 5:**
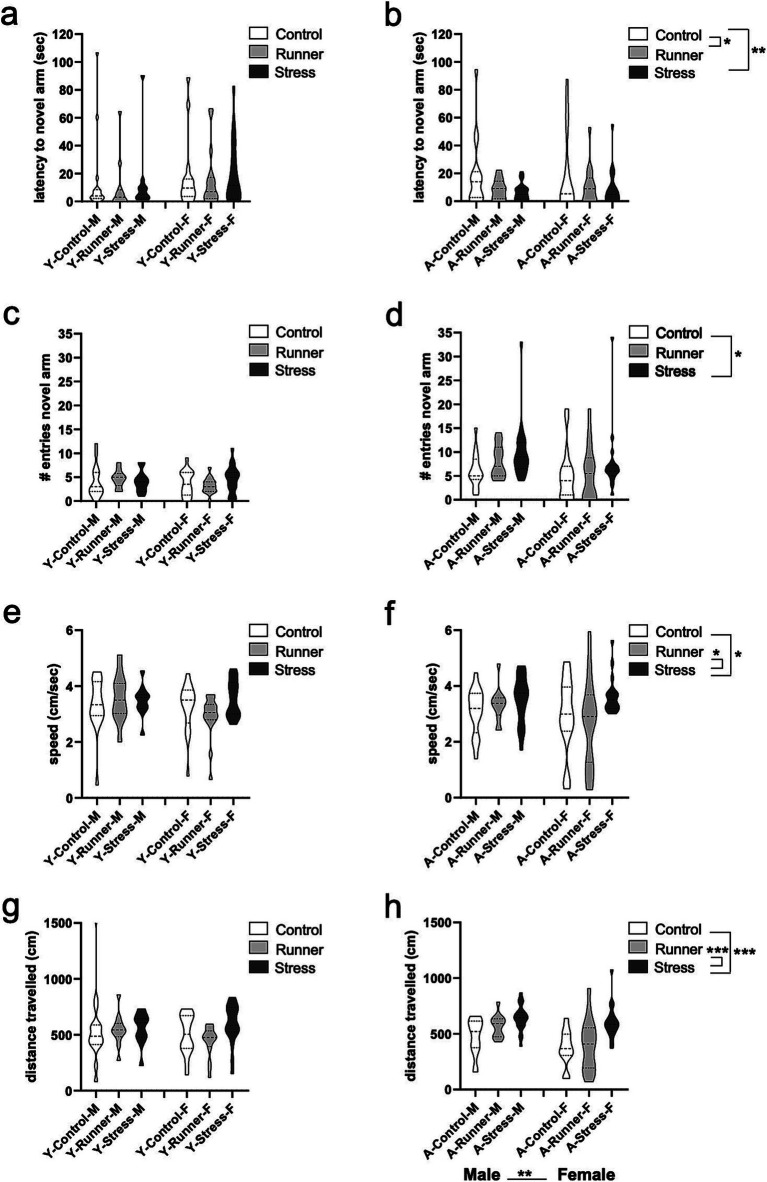
Y-maze recognition memory and activity. The latency (sec) to enter a novel arm (**A**, young; **B**, aged) and number of entries into a novel arm of the Y-maze (YM) (**C**, young; **D**, aged). Aged stressed mice exhibit hyperactive behavior in the YM. Speed (cm/s) (**E**, young; **F**, aged) and distance travelled (cm) (**G**, young; **H**, aged) during the YM task. ^*^p < 0.05, ^**^p ≤ 0.01, and ^***^p < 0.001.

Rearing moderated memory performance in the Y-maze in aged mice, with both running and stress-rearing enhancing preference for the novel arm of the Y-maze after the familiarization trial. When assessing the latencies to enter the novel arm of the maze, a significant main effect of rearing was observed [*F*_(2,98)_ = 4.033, *p* = 0.021, *η*^2^*_p_* = 0.078], with runners (*p* = 0.035) and stressed mice (*p* = 0.008) having shorter latencies to enter the novel arm relative to aged control mice (Figure 5B). This same main effect of rearing was not observed in young mice [*F*_(2,84)_ = 0.246, *p* = 0.782, *η*^2^*_p_* = 0.006; Figure 5A]. Enhanced memory for the novel spatial location in the Y-maze was also reflected as a marginal main effect of rearing on the total number of entries into the novel arm of the maze in aged mice [*F*_(2,98)_ = 3.179, *p* = 0.046, *η*^2^*_p_* = 0.061], with stressed mice (*p* = 0.011) entering the novel arm more often than controls (Figure 5D). These findings reflect the hyperactivity observed in aged stressed mice. The same preference for the novel arm of the Y-maze was not observed in young mice (all *p*’s >0.05; Figure 5C). There were no other main effects of sex or rearing × sex interaction for recognition memory (all *p*’s >0.05).

Activity in the Y-maze was generally moderated by rearing condition in aged mice, but these effects were not observed in young mice. In aged mice, a main effect of rearing was evident for speed travelled in the Y-maze [*F*_(2,98)_ = 3.537, *p* = 0.033, *η*^2^*_p_* = 0.067], with stressed mice travelling faster than controls (*p* = 0.024) and runners (*p* = 0.017 Figure 5F). Similarly, a significant main effect of rearing emerged for distance travelled in the Y-maze [*F*_(2,98)_ = 15.432, *p* < 0.001, *η*^2^*_p_* = 0.240], with aged stressed mice exploring further distances than aged controls (*p* < 0.001) and runners (*p* < 0.001; Figure 5H). A main effect of sex was also observed [*F*_(2,98)_ = 10.525, *p* = 0.002, *η*^2^*_p_* = 0.097] with aged males exploring further distances than females. No rearing × sex interaction were observed (all *p*’s >0.05). These results suggest that prolonged exposure to chronic mild stress induces a hyperactive phenotype in aged mice. Young mice did not display similar rearing effects on speed [*F*_(2,84)_ = 0.988, *p* = 0.376, *η*^2^*_p_* = 0.023] (Figure 5E), or distance travelled in the Y-maze [*F*_(2,84)_ = 1.181, *p* = 0.312, *η*^2^*_p_* = 0.027] (Figure 5G), nor any main effect of sex or rearing × sex interaction (all *p*’s >0.05).

#### 3.3.2 Novel object recognition

**Figure 6:**
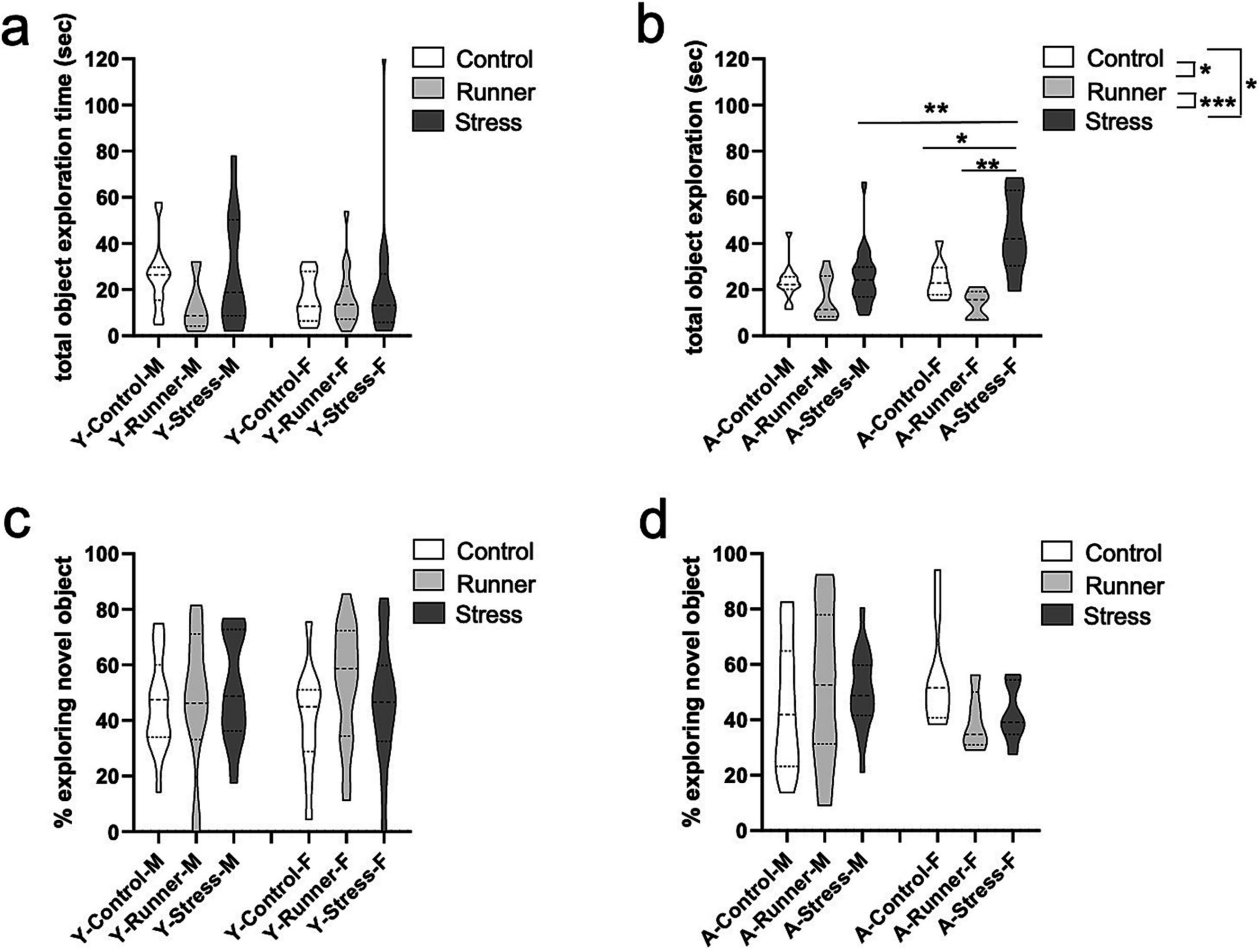
Novel object recognition performance. Aged stressed mice exhibit hyperactive behavior in the novel object recognition (NOR) task. Total explorations time (sec) (**A**, young; **B**, aged) and proportion of time (%) exploring the novel object (**C**, young; **D**, aged) during the NOR task. ^*^p < 0.05, ^**^p ≤ 0.01, and ^***^p < 0.001.

We assessed exploratory behavior in the NOR task. Here too, aged mice demonstrated a hyperactive phenotype, with a main effect of rearing condition on total exploration duration [*F*_(2,59)_ = 13.984, *p* < 0.001, *η*^2^*_p_* = 0.322]. Stressed mice explored both objects significantly longer than control (*p* = 0.042) and runner (*p* < 0.001), while runners explored significantly less than controls (*p* = 0.022; Figure 6B). A significant rearing × sex interaction was revealed for aged mice [*F*_(2,59)_ = 4.841, *p* = 0.011, *η*^2^*_p_* = 0.141], with the effect driven by significantly longer exploration times by female stressed mice when compared with sex-matched controls [*t*_(13)_ = −2.979, *p* = 0.011, *d* = 1.145] and runners [*t*_(9)_ = −3,603, *p* = 0.006, *d* = 2.283] (Figure 6B). A significant sex difference also emerged for stressed aged mice, with females exhibiting higher exploratory behavior than males [*t*_(23)_ = −2.832, *p* = 0.009, *d* = 1.199] (Figure 6B). Together, these findings confirm a hyperactive phenotype in aged stressed female mice that is not observed in males. Young mice did not display similar rearing effects on total exploration time [*F*_(2,73)_ = 1.409, *p* = 0.251, *η*^2^*_p_* = 0.038] (Figure 6A), a main effect of sex [*F*_(1,73)_ = 0.699, *p* = 0.406, *η*^2^*_p_* = 0.010], nor an interaction [*F*_(2,73)_ = 0.536, *p* = 0.587, *η*^2^*_p_* = 0.015] (Figure 6A).

Despite increased exploratory behavior in aged mice, no differences in recognition memory performance were observed for either young or aged mice [young: rearing (*F*_(2,73)_ = 0.784, *p* = 0.460, *η*^2^*_p_* = 0.021); sex (*F*_(1,73)_ = 0.074, *p* = 0.786, *η*^2^*_p_* = 0.001); rearing × sex interaction (*F*_(2,73)_ = 0.453, *p* = 0.637, *η*^2^*_p_* = 0.012) (Figure 6C). Aged: rearing (*F*_(2,59)_ = 0.292, *p* = 0.748, *η*^2^*_p_* = 0.010), sex (*F*_(1,59)_ = 0.421, *p* = 0.519, *η*^2^*_p_* = 0.007), rearing × sex interaction (*F*_(2,59)_ = 1.901, *p* = 0.159, *η*^2^*_p_* = 0.061) (Figure 6D)], indicating that neither stress nor running affected the percent of time spent exploring the novel object.

#### 3.3.3 Spatial water maze

**Figure 7:**
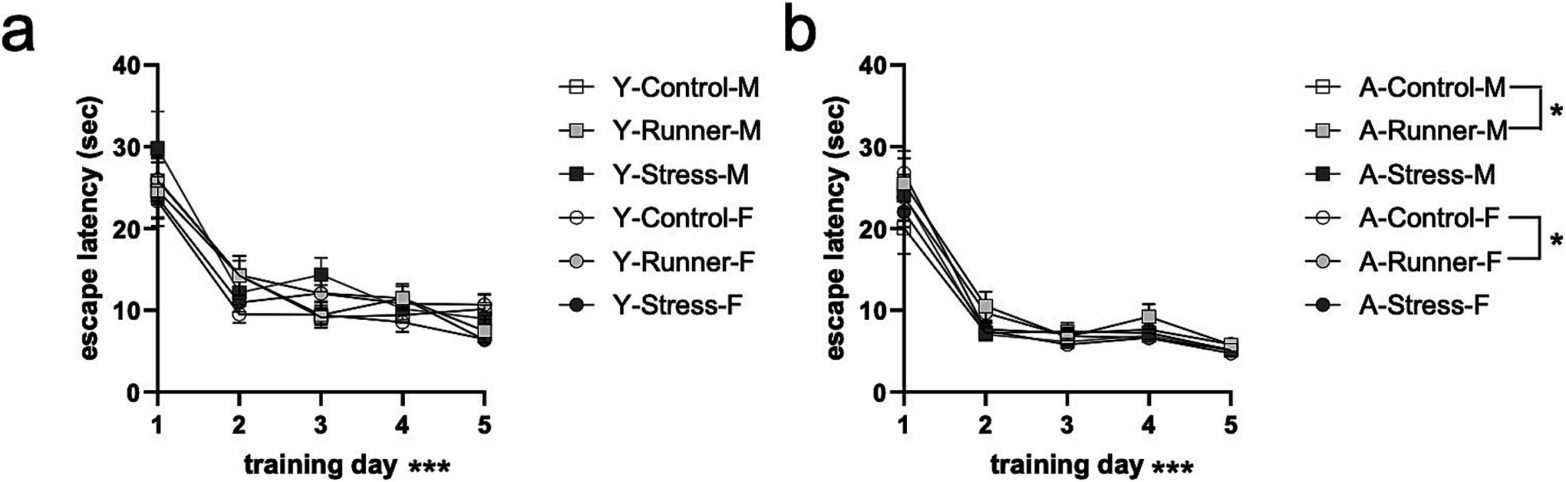
Spatial water maze. Escape latency (sec) decreased across 5 training days (**A**, young; **B**, aged). ^*^p < 0.05 and ^***^p < 0.001.

A repeated measures ANOVA with water maze training day (day 1–5) as a within-subjects factor and rearing and sex as between-subjects factors showed main effects of day [*F*_(1,76)_ = 124.143, *p* < 0.001, *η*^2^*_p_* = 0.620] for young mice, with post-hoc tests finding that escape latencies decreased across testing days (*p*’s <0.005, corrected for multiple comparisons; Figure 7A). No main effect of rearing [*F*_(2,76)_ = 1.159, *p* = 0.319, *η*^2^*_p_* = 0.030], sex [*F*_(1,76)_ = 2.219, *p* = 0.140, *η*^2^*_p_* = 0.028], or a rearing × sex interaction [*F*_(2,76)_ = 2.442, *p* = 0.094, *η*^2^*_p_* = 0. 060] was identified. Aged mice similarly showed a main effect of day [*F*_(1,77)_ = 244.966, *p* < 0.0001, *η*^2^*_p_* = 0.761], with escape latencies decreasing across testing days (*p*’s <0.001; Figure 7B). A marginal main effect was observed for rearing [*F*_(2,77)_ = 3.138, *p* = 0.049, *η*^2^*_p_* = 0.075], with aged runners having overall faster escape latencies than controls (*p* = 0.16) (Figure 7B). No main effect of sex [*F*_(1,77)_ = 0.198, *p* = 0.657, *η*^2^*_p_* = 0.003] nor rearing × sex interaction [*F*_(2,77)_ = 0.372, *p* = 0.2691, *η*^2^*_p_* = 0.010] were observed. These findings indicate that prolonged running confers mild protective effects on spatial memory in aged mice relative to sedentary control-reared mice, a finding which is not evident in young mice exposed to only one month of running.

#### 3.3.4 Context and tone fear memory

**Figure 8:**
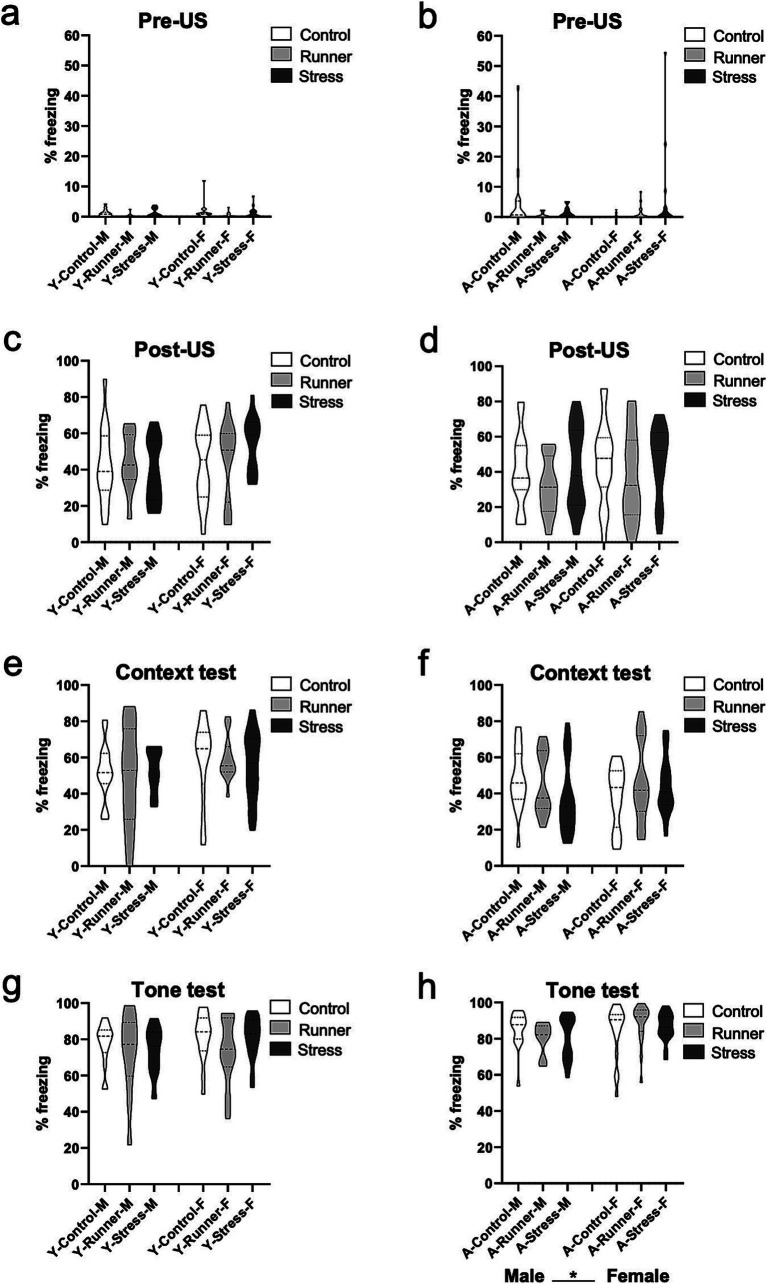
Fear conditioning. Rearing conditions did not affect fear behavior in young or aged mice. No differences were observed in the conditioning chamber prior to shock presentation (pre-US) (**A**, young; **B**, aged), and all groups displayed comparable levels of freezing following the final shock presentation during the conditioning trial (post-US) (**C**, young; **D**, aged). Twenty-four hours later, mice in all rearing conditions displayed comparable levels of freezing behavior (% freezing) during the 3 min context test phase (**E**, young; **F**, aged), and during the 3 min tone test phase (**G**, young; **H**, aged). ^*^p < 0.05.

In a test of context and cued tone fear memory, no group differences for rearing were observed for either age group. Prior to the presentation of shock, both male and female young and aged mice from all rearing conditions exhibited comparable levels of activity in the conditioning chamber [young: rearing (*F*_(2,82)_ = 2.157, *p* = 0.122, *η*^2^*_p_* = 0.050); sex (*F*_(1,82)_ = 0.261, *p* = 0.611, *η*^2^*_p_* = 0.003) (Figure 8A); aged: rearing (*F*_(2,97)_ = 1.060, *p* = 0.351, *η*^2^*_p_* = 0.021); sex (*F*_(1,97)_ = 0.001, *p* = 0.980, *η*^2^*_p_* = 0.000) (Figure 8B)], indicating that neither stress nor running affected baseline levels of anxiety-like behavior during the task. Following the presentation of the three foot shocks during the conditioning trial, all groups exhibited equivalent levels of post-shock freezing [Young: rearing (*F*_(2,82)_ = 0.325, *p* = 0.723, *η*^2^*_p_* = 0.008); sex (*F*_(1,82)_ = 1.862, *p* = 0.176, *η*^2^*_p_* = 0.022) (Figure 8C). Aged: rearing (*F*_(2,97)_ = 1.747, *p* = 0.180, *η*^2^*_p_* = 0.035); sex (*F*_(1,97)_ = 0.983, *p* = 0.324, *η*^2^*_p_* = 0.010) (Figure 8D)]. Twenty-four hours later, all groups exhibited comparable levels of freezing during the context memory test [Young: rearing (*F*_(2,82)_ = 0.033, *p* = 0.967, *η*^2^*_p_* = 0.001); sex (*F*_(1,82)_ = 2.283, *p* = 0.135, *η*^2^*_p_* = 0.027) (Figure 8E). Aged: rearing (*F*_(2,97)_ = 0.500, *p* = 0.608, *η*^2^*_p_* = 0.010); sex (*F*_(1,97)_ = 0.038, *p* = 0.846, *η*^2^*_p_* = 0.000) (Figure 8F)], and during the subsequent cued tone fear memory test [Young: rearing (*F*_(2,82)_ = 1.319, *p* = 0.273, *η*^2^*_p_* = 0.031); sex (*F*_(1,82)_ = 1.277, *p* = 0.262, *η*^2^*_p_* = 0.015) (Figure 8G). Aged: rearing (*F*_(2,97)_ = 0.159, *p* = 0.853, *η*^2^*_p_* = 0.003); sex (*F*_(1,97)_ = 4.209, *p* = 0.043, *η*^2^*_p_* = 0.042), (Figure 8H). All rearing × sex interactions *p*’s >0.05]. These findings indicate that acute or prolonged stress or running did not impair mice’s ability to form a robust fear memory across the lifespan, nor did it enhance the sensitivity or reactivity of the mice to an aversive shock stimulus.

### 3.4 Hippocampal BrdU labelling

**Figure 9:**
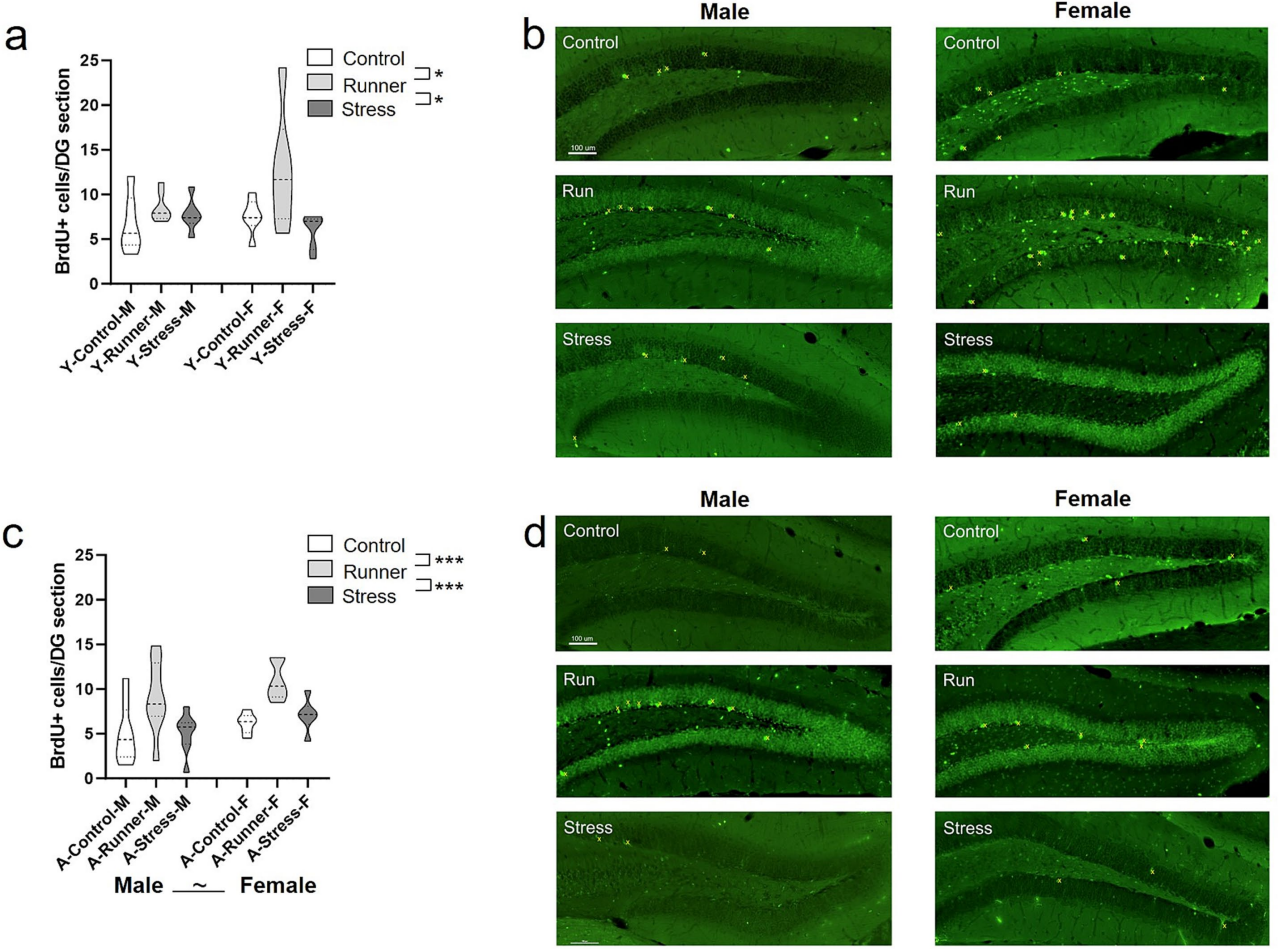
Hippocampal neurogenesis. Running enhanced rates of hippocampal BrdU labelled cells in both young and aged mice. Mean number of BrdU^+^ neurons per section in the dentate gyrus of the hippocampus (**A**, young; **C**, aged). Representative BrdU^+^ cell labelling per condition (**B**, young; **D**, aged; top: control; middle: runners; bottom: stress). BrdU^+^ cells are marked in yellow. Scale bar represents 100 μm. ^~^*p* = 0.059, ^*^p < 0.05, and ^***^p ≤ 0.001.

Following rearing, rates of hippocampal cell division were assessed using end-point BrdU labelling in the dentate gyrus. For both young and aged mice, those reared under running conditions had enhanced rates of BrdU-labelled cells. In young mice, a main effect of rearing was observed [*F*_(2,32)_ = 5.318, *p* = 0.010, *η*^2^*_p_* = 0.249], with significantly higher rates of post-rearing BrdU-labelled cells in young runners relative to controls (*p* = 0.020) and stressed mice (*p* = 0.012) (Figures 9A,B)). No main effects emerged for sex [*F*_(2,32)_ = 0.157, *p* = 0.695, *η*^2^*_p_* = 0.005], nor a rearing × sex interaction [*F*_(2,32)_ = 1.232, *p* = 0.305, *η*^2^*_p_* = 0.071] following 1 month of rearing. A main effect of rearing was also observed in aged mice [*F*_(2,36)_ = 10.599, *p* < 0.001, *η*^2^*_p_* = 0.371], with significantly higher rates of post-rearing BrdU-labelled cells in aged runners relative to controls (*p* < 0.001) and stressed mice (*p* = 0.001) (Figures 9C,D), while the main effect of sex [*F*_(1,36)_ = 3.799, *p* = 0.059, *η*^2^*_p_* = 0.095] was non-significant. No rearing × sex interaction was observed in aged mice [*F*_(2,36)_ = 0.91, *p* = 0.913, *η*^2^*_p_* = 0.005]. Together, running robustly enhanced survival of hippocampal BrdU-labelled cells across the lifespan in both sexes, while both short and long-term exposure to chronic mild stress did not significantly suppress hippocampal BrdU-labelled cells relative to sedentary control rearing.

## 4 Discussion

In line with previous findings, we found that voluntary running increased hippocampal cell division in both young and aged mice (Epp et al., 2021; Pereira et al., 2007; van Praag et al., 1999a, 1999b, 2005). While previous analyses of sex differences in rates of cell division and survival in young rats identified rates of attrition of newborn hippocampal neurons are higher in males than in females (Yagi et al., 2020), here, no differences in rates of Brdu^+^ cell survival 1-month post-labelling were observed in young mice, although BrdU labelling alone cannot identify cell type (neuron or glial). However, sex differences were evident in aged mice, with females retaining slightly higher survival of late-adult newborn cells, regardless of environmental stressors or exercise. Sex-specific effects of stress were not evident in the BrdU^+^ cell survival of young or aged mice. Previous reports in young adult rats exposed to 2 weeks of restraint stress did show evidence of a sex-specific reduction of survival of BrdU^+^ hippocampal neurons in females (Hillerer et al., 2013). It may be that the high levels of restraint stress-induced corticosterone in females relative to males contributed to the sex-differences in cell survival (Hillerer et al., 2013). While corticosterone measures were not obtained in the present study, it is possible that the varied mild stressors employed for 1 month in young mice were not sufficient to induce corticosterone mediated suppression of hippocampal proliferation and survival. To thoroughly characterize the cumulative and progressive impact of chronic stress on the brain and behaviors into aging, it will be informative for future investigations to repeatedly assesses additional biochemical and physiological variables including hormone and corticosterone levels, as well as a longitudinal behavioral assessment rather than using only end-point behavior and BrdU measures. Given normal age-related declines in neural stem cells and hippocampal neurogenesis neurotrophic factors, and brain volume which may be moderated by running behavior into late-adulthood (Connolly et al., 2022; Gao et al., 2023; Gil-Mohapel et al., 2013; Zocher and Toda, 2023), repeated *in-vivo* assessments of brain volume and structural changes over time, as well as cell specific measures of neural plasticity (DCX, Ki67, NeuN, BDNF, GFAP, IBA1) will be essential to identifying the neural mechanisms mediating sex-specific differences in response to environmental factors across the lifespan. Previous investigation of granule cell and molecular layer volumes in the dentate gyrus also identified age-related reductions associated with a smaller pool of precursor cells (Olariu et al., 2007), and fewer synaptic inputs into the granule cells (Geinisman and Bondareff, 1976; Geinisman et al., 1992), rather than being attributable to a gross reduction in the number of granule cells or the dendritic complexity of granule cells (Chawla and Barnes, 2007; Morgenstern et al., 2008). The factors contributing to potential age-related differences in hippocampal cell layer composition and morphology have not yet been systematically investigated between sexes, and highlight an avenue for further investigation in order to identify the factors that are most susceptible to the cumulative effects of stress or exercise into late-adulthood.

Studies involving prolonged periods of voluntary wheel running using single housing introduce a confounding stressor associated with extended periods of social isolation (Barha et al., 2017b). The environmental enrichment associated with group exercise is an important mediator of running-related enhancements of hippocampal neurogenesis (Stranahan et al., 2006). Given the manipulation of periodic social isolation as one of the stressors in the stress rearing condition, we deliberately chose to group-house running-reared mice to avoid introducing confounding stress induced by single housing. Consistent with a landmark study using group-running into late-adulthood that identified increased hippocampal BrdU^+^ cells (Kronenberg et al., 2006), the use of group housing during the 15-month running period limited the ability to track individual running activity of each mouse. As a result, directly correlating running behavior with each animal’s results are not possible, limiting the identification of a dose-dependent relationship between rates of running behavior and brain and behavioral measures between sexes. Previous investigation in young male rats exposed to voluntary running for 30 days did find a relationship between rates and intensity of running behavior and BrdU^+^ cell survival, with moderate running activity (4–8 h/day) producing the greatest benefit to cell survival. Interestingly, they found a negative correlation between distance run and BrdU^+^ cell survival, suggesting that prolonged, excessive running behavior may be detrimental to the survival of newly generated hippocampal cells (Nguemeni et al., 2018). In the aged brain, there is evidence that longer durations of running are required to induce a neurogenic boost, with higher rates of hippocampal neurogenesis observed after 7, but not 2, weeks of running in aged male mice (Connolly et al., 2022). Given the prolonged testing period following rearing, it is possible that the rated of BrdU^+^ cell survival observed in the present study reflect a more cumulative response to the interventions, rather than the acute effects of stress or exercise which might have been captured by testing immediately following the rearing interventions.

We observed that prolonged exercise (15 months) was associated with quicker acquisition of a spatial learning task compared to stressed and sedentary control aged mice, while acute (1 month) mild stress or exercise was not sufficient to significantly influence memory performance in any task in young mice. Despite an enhancement of spatial memory performance in the water maze, no other enhancements of memory were observed in aged mice, suggesting that running throughout the lifespan induced only a modest cumulative protective effect on memory. Though no sex-specific differences in memory performance were observed in response to running or stress exposure in the present study, increasing stress during a spatial water maze task by using cold water identified sex-specific search strategies, in which females used more variable search strategies relative to males (O’Leary et al., 2022), suggesting that the timing of exposure and type of stress are important factors to consider when assessing stress-induced sex differences in brain and behavioral activity. In the present study, stress did not differentially affect the acquisition of spatial memory across training days in either young or aged mice, but a more nuanced evaluation of search strategies may have identified potential sex differences associated with acute or prolonged stress exposure and performance in the water maze.

Methodological variability in common chronic stressor models depends on whether the intent is to model depression, post-traumatic stress, or other affective and mood disorders (Duman and Monteggia, 2006; Schöner et al., 2017; Antoniuk et al., 2019). The majority of chronic stress models have been optimized using exclusively male mice, and resultantly may not adequately model stress-related behaviors experienced by females (Lopez and Bagot, 2021). The decision to use multiple mild stressors in the present study was motivated by the goal of mimicking relevant everyday stressors in mouse’s natural environment (occasional food or water deprivation, proximity to predator, periodic isolation, circadian disruption, soiled nesting area) to enhance the ecological validity of unpredictable daily stressors relevant to a rodent (Antoniuk et al., 2019).

Life-long running was associated with low body weight in aged males, but not females, while long-term stress exposure resulted in low body weights in both aged males and females. Low weight gain in aged male and female rodents has been previously observed following several weeks of restraint stress exposure (Bowman et al., 2006; Bloss et al., 2010). The behavioral manifestations of stress appear to vary across sex, with both young and aged females displaying a more anxious-like phenotype in the open field, relative to males. Previous findings following late-life restraint stress found that stress differentially increased anxiety-like behavior in the open field in males but not in females, while having no effect on overall activity levels (Bowman et al., 2006). Aged females also show an increased depressive-like phenotype compared to males, while no differences in sex were evident in younger mice. Hyperactivity and enhancements in anxiety-like behavior in aged mice were the most notable observations following prolonged stress exposure. In the open field, elevated plus maze, and Y-maze exploration tasks, aged stressed mice generally exhibited a hyperactive phenotype, which was not observed in young mice. This effect was especially pronounced in aged female stressed mice, consistent with prior observations of hyperactivity in aged females exposed to social isolation stress in late-adulthood (Sullens et al., 2021). Hyperactivity and impulsivity following prolonged exposure to stress has been commonly observed in young and aged rodents (Ashby et al., 2010; An et al., 2017; Strekalova et al., 2005; Schweizer et al., 2009), with some studies interpreting hyperlocomotion or hyperactivity as a symptom of chronic stress, potentially confounding typical behavioral measures such as the open field and the forced swim task in which low activity is thought to be indicative of anxiety-like and depressive-like behaviors (Strekalova et al., 2005, 2022). As noted by Strekalova et al. (2005), stress-induced hyperactivity may be exacerbated by strong illumination, as evident in the present study’s observed enhanced distance travelled in the open field by stressed mice, irrespective of age or sex, and high speed of exploration for aged males and females. In support of this interpretation, during the beam walk test where bright illumination is used at the starting point, the rapid escape latency observed in aged males and females may be reflective of a hyperactive phenotype following prolonged stress into late-adulthood. Interpreting the meaning behind the observed hyperactive behavior remains a significant challenge to rodent models of stress, anxiety-like and depression-like behaviors, with few studies directly considering the differential effects between biological sex (Lima et al., 2022).

In sum, we provide evidence supporting differential effects of exercise and chronic mild stress across the lifespan in male and female mice, and highlight the sensitivity of females to stress-induced increases in hyperactivity, anxiety-like and depressive-like behavior with age. A growing body of literature highlights the importance of considering sex as a biological variable when determining how healthy aging is differently impacted by modifiable lifestyle factors across the lifespan, and points to the need to develop models that explicitly include biological sex as a variable of interest using standardized models of chronic stress or enrichment and a standardized behavioral battery in order to improve the methodological rigor required to systematically investigate sex differences in aging models.

## Data Availability

The raw data supporting the conclusions of this article will be made available by the authors, without undue reservation.
