# Supplementary material for: Sex in aging matters: exercise and chronic stress differentially impact females and males across the lifespan
Source: Front Aging Neurosci. 2025 Jan 15;16:1508801. doi: 10.3389/fnagi.2024.1508801 (PMC11774976; doi:10.3389/fnagi.2024.1508801)
Supplement: Supplementary file 1 [file Table_1.DOCX]

**SUPPLEMENTAL MATERIALS**

**Table S1. Young and Aged Rearing Groups**

Number of mice per rearing condition.


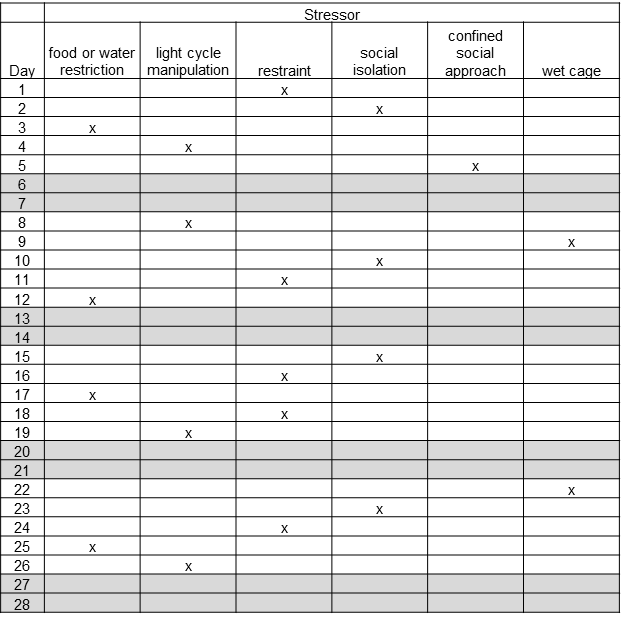


**Table S2. Sample of monthly stressor schedule.**

Maximum of five stressors per week. Food or water restriction (23 hr duration; maximum 3 non-consecutive sessions/week), light cycle manipulation (36 hr duration; maximum 2 non-consecutive sessions/week), restraint (1 hr duration; maximum 2 non-consecutive sessions/week), social isolation (8 hr duration; maximum 1 session/week), confined social approach (10 min duration; maximum 1 session/month), wet cage (8 hr duration; maximum 2 non-consecutive sessions/month).
